# Esophageal Cancer in a Mid-esophageal Diverticulum: A Case Report on Diagnostic Challenges and Treatment Outcomes

**DOI:** 10.7759/cureus.91299

**Published:** 2025-08-30

**Authors:** Garima Vats, Prajwal Chandrashekhara, Anuj, Kalale Radhakrishnan Bhagavan

**Affiliations:** 1 General Medicine, K S Hegde Medical Academy, Mangaluru, IND; 2 General Surgery, K S Hegde Medical Academy, Mangaluru, IND

**Keywords:** chemotherapy, esophageal diverticulum, esophageal malignancy, oncology, radiotherapy, squamous cell carcinoma

## Abstract

Esophageal diverticula are rare pouches that form in the esophageal lining. They occur infrequently in the general population and are more often seen in individuals with dysphagia. Among the types, mid-esophageal (parabronchial) diverticula are especially uncommon. These diverticula are usually asymptomatic and are often found incidentally during diagnostic procedures. Unlike distal esophageal diverticula, which may be linked to acid reflux, mid-esophageal types are thought to result from long-standing inflammation or mechanical forces acting on the esophagus. Although cancer arising in esophageal diverticula is uncommon, when it does occur, it may progress more rapidly due to structural weaknesses in the diverticular wall.

We present the case of a middle-aged man with progressive difficulty swallowing and a persistent dry cough. Endoscopy identified a large mid-esophageal diverticulum approximately 25 cm from the incisors, with irregular mucosal changes. Initial biopsies were non-diagnostic, showing granulation tissue. A contrast-enhanced CT scan later revealed a mass in the lower esophagus with signs of spread to the lungs. Bronchoscopy confirmed the diagnosis of squamous cell carcinoma. The patient underwent six cycles of chemotherapy and radiotherapy with a positive response. This case illustrates the challenges in diagnosing malignancy within an esophageal diverticulum and underscores the importance of thorough evaluation in symptomatic patients, as early detection can significantly influence treatment and outcomes.

## Introduction

An esophageal diverticulum is a rare condition characterized by an outpouching of the esophageal mucosa. It affects less than 1% of the general population and occurs in approximately 1% to 3% of individuals presenting with dysphagia [1-2]. A mid-esophageal diverticulum, also known as a parabronchial diverticulum, is typically located about 5-6 cm distal to the tracheal bifurcation [3]. The risk of malignancy in esophageal diverticula varies by location, with reported rates of 0.3-7.1% in pharyngeal, 1.8% in mid-esophageal, and 0.6% in epiphrenic diverticula [4]. A Japanese study identified the most common sites of esophageal diverticula as the pharyngeal esophageal region (Zenker’s diverticulum, 15.8%), the tracheal bifurcation (Rokitansky diverticulum, 73.0%), and the supradiaphragmatic region (11.2%) [5]. Mid-esophageal diverticula are relatively uncommon, often asymptomatic, and typically discovered incidentally. Unlike distal diverticula, which are frequently associated with gastroesophageal reflux disease, mid-esophageal diverticula are more commonly related to chronic mediastinal inflammation and traction forces exerted on the esophageal wall [1].

Pulsion diverticula are considered false diverticula because they lack a muscular layer and result from elevated intraluminal pressures, typically due to underlying esophageal motility disorders [6]. Due to the absence of the muscularis propria, malignancy in pulsion diverticula may infiltrate the adventitia and adjacent tissues more readily [7].

## Case presentation

A male patient in his early fifties presented with a six-month history of progressively worsening dysphagia to solids, which had intensified over the past month but improved with liquids. He also reported a persistent change in voice over the same six-month period and a dry cough for the past 25 days. Additionally, the patient recalled experiencing mild dysphagia and postprandial cough intermittently over the past three years, though these symptoms were previously less severe. There was no history of dyspnea, hemoptysis, or other gastrointestinal symptoms such as vomiting, abdominal pain, distension, diarrhea, or constipation. His medical history was significant for type II diabetes mellitus for the past 10 years, with no history of tobacco or alcohol use. There was no personal or family history of malignancy. General and systemic examinations were unremarkable.

**Figure 1:**
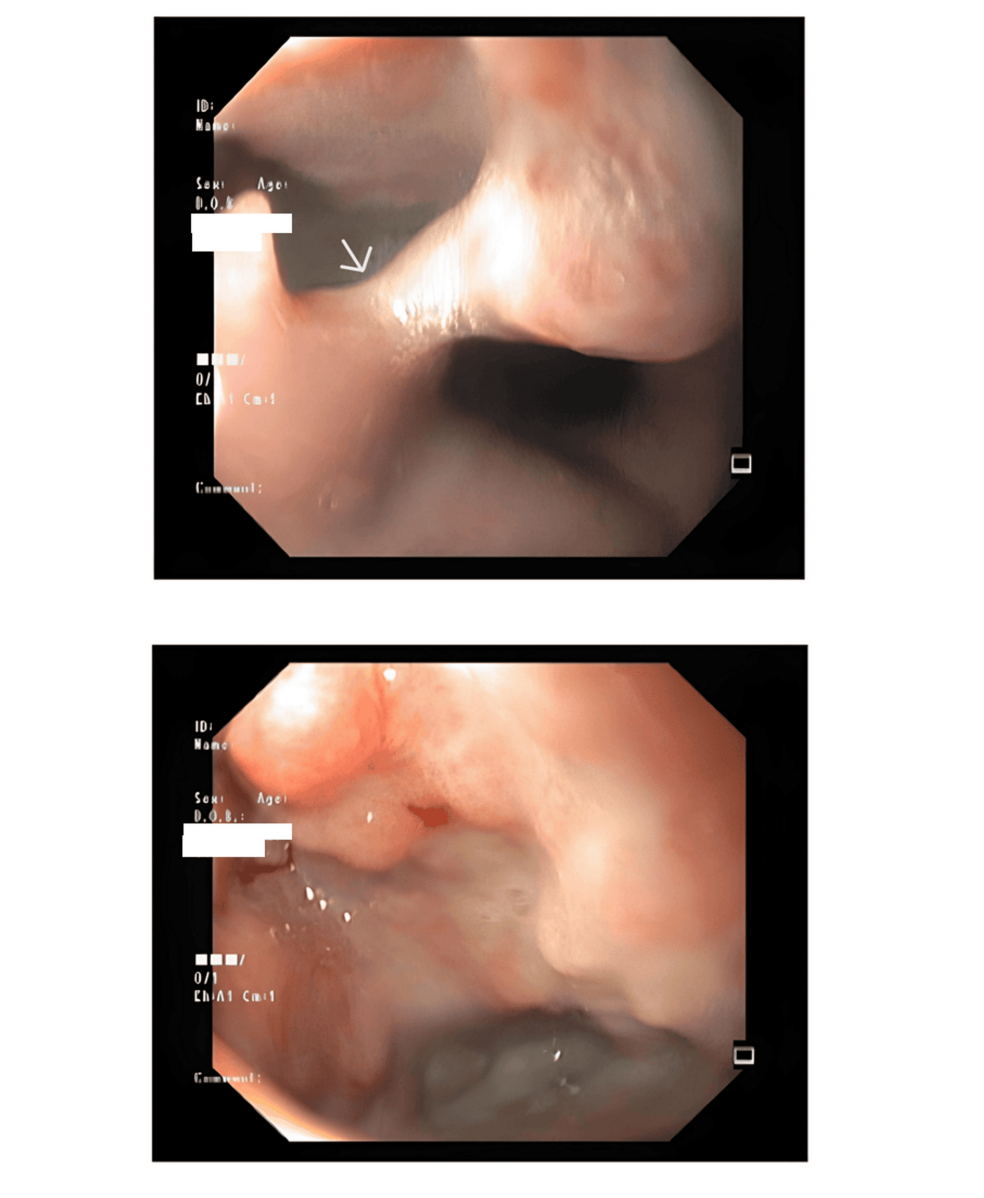
Esophagogastroduodenoscopy Esophagogastroduodenoscopy images depicted ulceroproliferative growth noted in the mid esophageal diverticula (marked by an arrow).

Given the progressive dysphagia, an upper gastrointestinal endoscopy (UGI scopy) was performed. This revealed a mid-esophageal diverticulum approximately 6-8 cm in length, located 25 cm from the incisors. The mucosal surface appeared irregular, prompting multiple biopsies. Histopathology revealed normal mucosa with granulation tissue (Figure 1).

**Figure 2:**
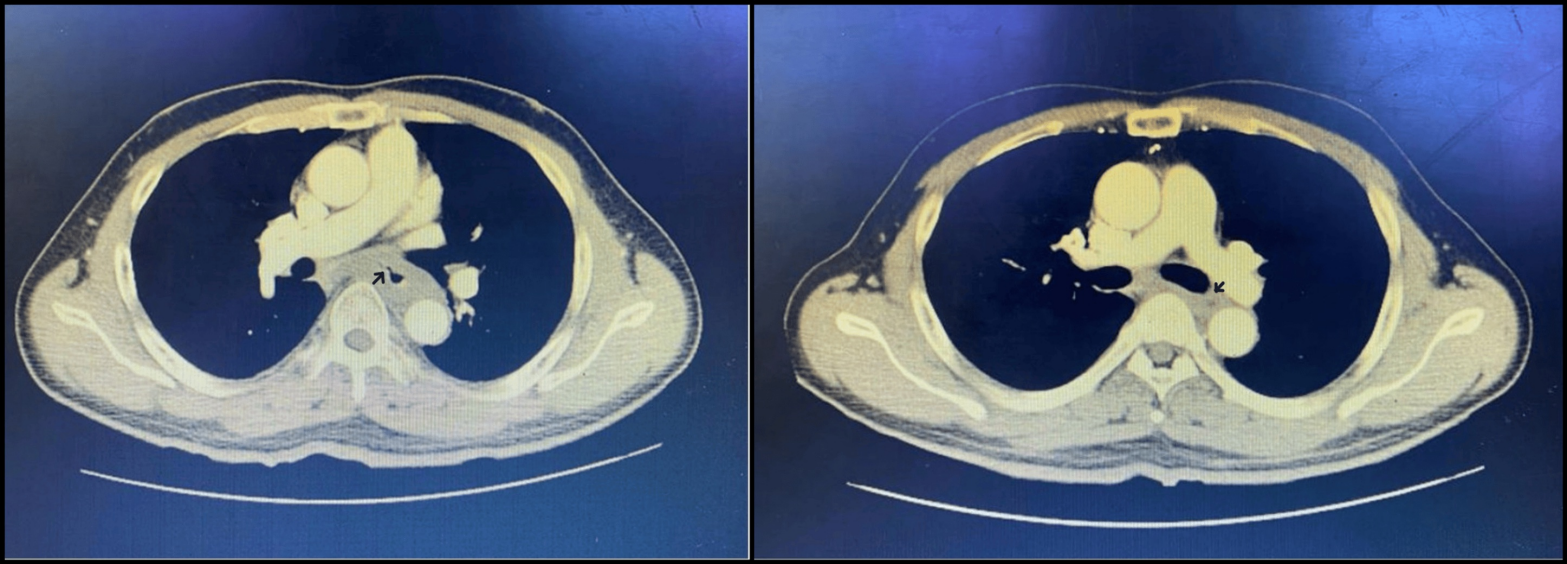
Contrast-enhanced CT (CECT) of the thorax CECT Thorax images depicting circumferential enhancing wall thickening of infracarinal thoracic oesophagus measuring a maximum thickness of 10 mm for a length of 7.6 cm, causing luminal narrowing. Indistinct fat planes with the left main bronchus, left atrium, arch of aorta, left main pulmonary artery, and descending thoracic aorta (marked by arrows).

In spite of the normal biopsy report, further evaluation was done based on clinical suspicion; a contrast-enhanced CT (CECT) scan of the thorax was performed. The scan revealed circumferential wall thickening in the infracarinal thoracic esophagus (10 mm thick and 7.6 cm long), causing luminal narrowing. Periesophageal fat stranding, loss of mural stratification, and indistinct fat planes with adjacent vital structures were noted. The esophageal thickening extended into the prevertebral space and encroached on surrounding vascular and bronchial structures. Additionally, metastatic deposits were identified in both lungs, strongly suggesting advanced malignancy (Figure 2).

**Figure 3:**
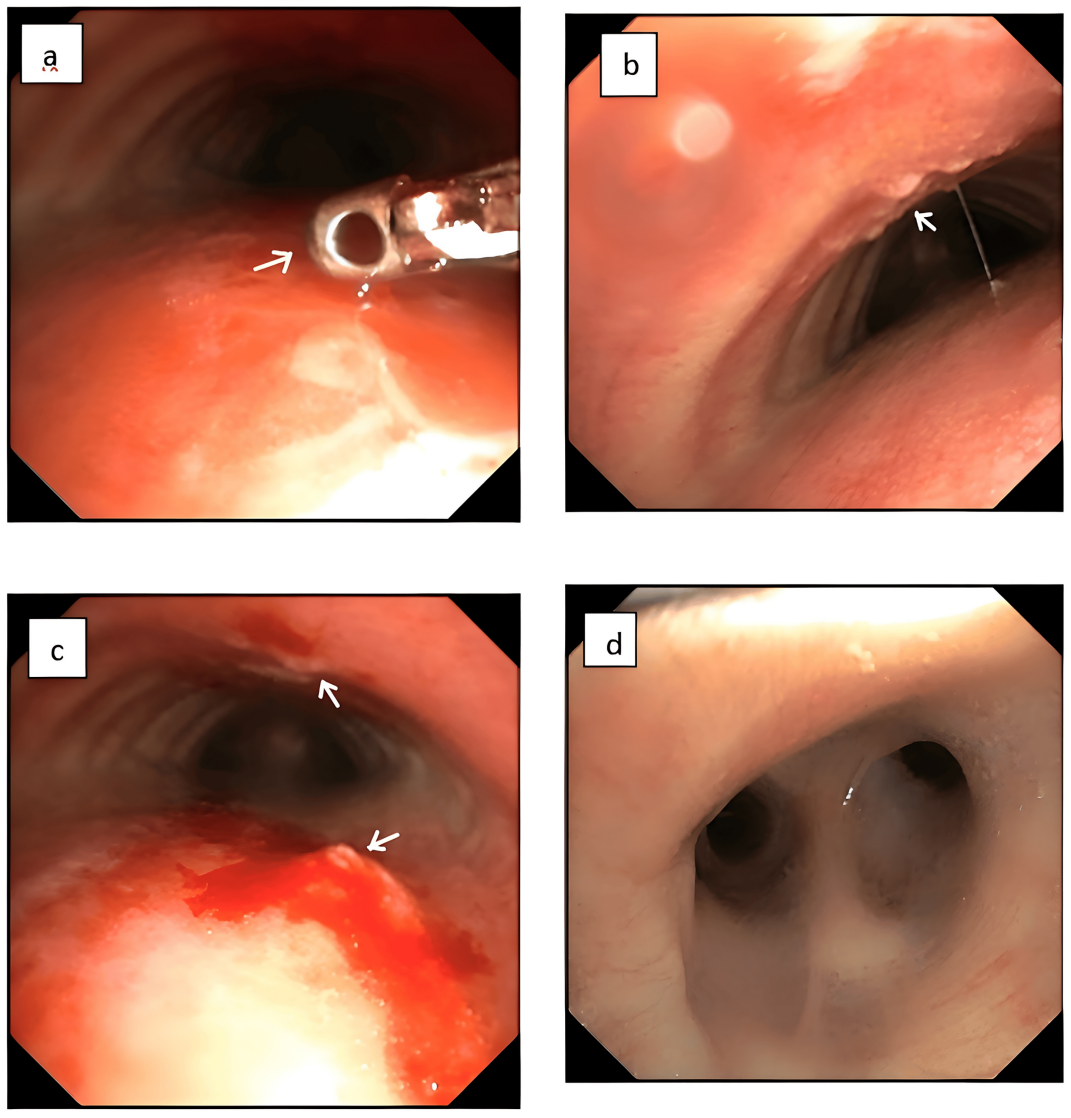
Bronchoscopy Bronchoscopy images: a. and c. Infiltrative growth seen in both anterior and posterior walls of the trachea (marked by arrows); b. mucosal bulge seen at the distal end of the left main bronchus (marked by an arrow); d. the main carina appears normal.

Despite repeated biopsies showing only granulation tissue, the patient’s persistent symptoms, the endoscopic appearance of the lesion, and CT findings heightened clinical suspicion for malignancy. A bronchoscopy was performed to assess for airway involvement. This revealed infiltrative growths on the anterior and posterior walls of the trachea, along with a mucosal bulge at the distal end of the left main bronchus. Brush cytology taken during bronchoscopy and evaluated with rapid on-site evaluation (ROSE) was suggestive of squamous cell carcinoma originating from the esophagus with tracheal infiltration (Figure 3).

Subsequent histopathology confirmed moderately differentiated squamous cell carcinoma. Based on imaging and histopathological findings, a final diagnosis of metastatic infracarinal esophageal squamous cell carcinoma (T4aN3M1) was made.

The patient was initiated on treatment with three cycles of neoadjuvant chemotherapy (paclitaxel and carboplatin) followed by radical radiotherapy. A repeat CECT thorax revealed significant tumor reduction and resolution of the mucosal irregularity within the diverticulum. The patient subsequently completed three more cycles of chemotherapy and received 30 fractions of external beam radiation (54 Gy) over six weeks, with a favorable clinical response. 

Differential diagnostic reasoning

The patient presented with dysphagia, hoarseness of voice, and cough, raising clinical suspicion for esophageal malignancy and prompting an upper gastrointestinal (UGI) endoscopy. Endoscopic evaluation revealed a mid-esophageal diverticulum with irregular mucosa, raising concern for carcinoma arising within the diverticulum. To further assess the extent of disease, a CECT of the thorax was performed, which demonstrated esophageal wall thickening, loss of fat planes with adjacent mediastinal structures, and pulmonary metastases--findings suggestive of advanced malignancy. Endoscopic biopsies, however, were inconclusive, showing only granulation tissue. Despite this, the patient's age, symptoms, and radiologic findings maintained a high index of suspicion for malignancy. Subsequent bronchoscopy revealed tracheal infiltration, and cytological and histopathological analysis confirmed squamous cell carcinoma. This case highlights the diagnostic challenges of malignancy arising within an esophageal diverticulum, particularly when initial biopsies are non-diagnostic, and underscores the importance of a high degree of clinical suspicion and a multimodal approach to diagnosis and management.

Treatment

Treatment Rationale and Dose Calculations

The patient was planned for combined modality treatment with radiotherapy and chemotherapy (Table 1).

**Table 1:** Treatment phases with details

Treatment Phase	Details
Neoadjuvant Chemotherapy	Three cycles administered at 3-week intervals
Chemotherapy Regimen	Paclitaxel: 260 mg in 1 pint normal saline with ondansetron, infused over 3 hours - Carboplatin: 450 mg in 5% dextrose IV over 3 hours
Response to Initial Treatment	Significant tumor reduction and resolution of irregular mucosa in the diverticulum after neoadjuvant therapy followed by radical radiotherapy
Subsequent Chemoradiotherapy	Three additional cycles of chemotherapy using the same dosing schedule
Radiotherapy Details	30 fractions of external beam radiotherapy, delivering a total of 54 Gy over six weeks

Radiotherapy: Three-dimensional conformal radiotherapy (3D-CRT) was delivered to a total dose of 54 Gy in 30 fractions over 6 weeks (1.8 Gy per fraction, 5 fractions per week).

Chemotherapy: Concurrent systemic therapy with paclitaxel and carboplatin was administered. Based on a body surface area (BSA) of 1.6 m², the recommended dose of paclitaxel (175 mg/m²) corresponded to 280 mg, and an adjusted dose of 260 mg was given [8]. The carboplatin dose was calculated using the Calvert formula, with a glomerular filtration rate (GFR) of 67.2 mL/min. For a target area under the curve (AUC) of 5, as recommended in standard regimens [8], the calculated dose was approximately 461 mg, and the patient received 450 mg.

Outcome and follow-up

The patient received three cycles of neoadjuvant chemotherapy with paclitaxel and carboplatin, followed by reassessment showing a reduction in esophageal wall thickening and lung nodules. He subsequently underwent radical radiotherapy with significant tumor regression, then 3 additional chemotherapy cycles and 54 Gy radiation over 6 weeks.

Four months post-treatment, positron emission tomography (PET)-CT showed no residual esophageal lesion but revealed metastatic cervical lymphadenopathy and lung nodules. He was started on oral gefitinib 250 mg on alternate days, which he has continued for the past year. A repeat PET-CT four months ago showed mild regression of cervical nodes but no change in lung nodules. Hence, according to RECIST criteria (RECIST Working Group), despite complete resolution of the primary lesion, the lack of response in metastatic lung lesions classifies the case as stable disease (SD). Nonetheless, this still indicates a relatively favorable prognosis compared to typical outcomes in esophageal carcinoma [9].

## Discussion

Esophageal diverticula, though rare, have become a focus of surgical interest over the past two decades. The risk of malignancy is higher in pulsion diverticula compared with true diverticula because of their anatomical differences [4]. Chronic irritation and inflammation, often caused by food stasis within the diverticulum, are key factors contributing to malignant transformation [10]. Esophageal diverticular carcinoma frequently presents at an advanced stage since its symptoms, such as dysphagia, regurgitation, or halitosis, are often attributed to the diverticulum itself rather than to malignancy [11]. This makes both the need for early diagnosis and the difficulty of achieving it particularly critical. Barium esophagography remains a useful tool for initial evaluation, but UGI scopy has gained prominence over time, as it allows for biopsy and definitive exclusion of malignancy [12]. However, esophagoscopy carries a risk of perforation, and manometry should be performed first to rule out motility disorders [13]. In selected cases, bronchoscopy can provide important additional information, particularly for detecting tracheal infiltration and confirming malignancy. Although long-term survival has been reported in some cases, such as Dionigi et al. [14], the overall prognosis remains poor for patients with esophageal diverticula complicated by malignancy. This highlights the importance of strict surveillance and follow-up, with any mucosal changes--ulceration, thickening, or discoloration--biopsied without delay. If cancer is confirmed, management should follow standard esophageal cancer protocols, including surgery, endoscopic therapy, or chemoradiation, depending on stage [15].

Table 2 summarizes recently reported cases of esophageal diverticula with squamous cell carcinoma, including mid-esophageal pulsion diverticula, together with their clinical details and management. Notably, across these reports, all patients were non-metastatic at presentation and underwent local resection, either endoscopic submucosal dissection (ESD) for superficial lesions or diverticulectomy for Zenker’s and larger diverticula. Outcomes were favorable in the short term, with disease-free follow-up where available. These findings suggest that when staging confirms mucosal disease without nodal or distant spread, simple resection (ESD or surgical diverticulectomy) is both appropriate and effective.

**Table 2:** Recently reported cases of esophageal diverticula with squamous cell carcinoma, including mid-esophageal pulsion diverticula, summarized with key clinical details and management

Article (Year)	Size of Diverticulum	Extent of Carcinoma at Diagnosis	Metastatic Lesions Present	Treatment Given	Outcome	Reference Number
Fukuda et al., 2023	10 cm	cT1a-EP/LPM N0 M0 (in situ, 20mm lesion), pTis-EP, ly0, v0, R0	none	Surgical resection (diverticulectomy)	Discharged, no complications	[15]
Zhang et al., 2023	Shallow pulsion diverticulum	28 mm superficial esophageal cancer in the middle thoracic esophagus	none	Endoscopic submucosal dissection (ESD) followed by a temporary covered esophageal stent	Discharged, no complications	[16]
Polit et al., 2022	5.0 × 4.4 × 2.4 cm diverticulum	3.2 × 2.5 × 1 cm, exophytic, ulcerated, involving the full esophageal wall, abutting serosa. Moderately differentiated squamous cell carcinoma on microscopy, margins negative	none	Open surgical diverticulectomy (Zenker resection) followed by hematology-oncology reference	Not stated (but authors note that after simple diverticulectomy with clear margins, patients are expected to remain disease-free)	[17]
Fu et al., 2017	Large epiphrenic diverticulum just above the esophagogastric junction	Intraepithelial squamous cell carcinoma confined to lamina propria, no lympho-vascular invasion, margins negative.	none	Endoscopic submucosal dissection (ESD)	Uneventful hospital course. No local recurrence or metastasis at 42-month follow-up.	[18]
Tanaka et al., 2015	Shallow pulsion diverticulum, ~3 × 2 cm	Macroscopic type 0-Ⅱc, half circumference of the middle thoracic esophagus. Squamous cell carcinoma lesion confined to epithelium or lamina propria mucosa (NBI/IPCL).	none	Endoscopic submucosal dissection (ESD). Perforation occurred- repaired with clips + detachable snare	Mediastinal and subcutaneous emphysema, fever, and leukocytosis all resolved by day 7. Discharged in good condition.	[19]

## Conclusions

Although prognosis is generally poor for advanced esophageal diverticular carcinoma due to delayed diagnosis and the thin diverticular wall that allows rapid invasion, recent non-metastatic cases show that early detection and localized resection can lead to favorable outcomes. Symptoms may be mistaken for the diverticulum itself, leading to late detection. Regular endoscopic surveillance with prompt biopsy of suspicious changes is essential. When cancer is identified, treatment should follow standard esophageal cancer protocols. Vigilant monitoring and early intervention can markedly improve outcomes. 

## Appendices

Patient’s perspective

I have had health issues for 3 years. When I used to eat, I would have difficulty swallowing and would cough. I consulted a local hospital near me and was told that I have gastric issues. When I went to my friend’s son’s marriage 2 years back, I was unable to swallow well even with water. For the next 1.5 years, my symptoms were relatively stable, with little difficulty in swallowing. Six months ago, my symptoms suddenly worsened, and I was unable to swallow most regular food, especially solids. Hence, I came to visit a higher centre where I actually got my correct diagnosis. Initially, my doctor here also gave me some tablets for gastric problems and asked me to come back and get an endoscopy done if the problem persisted. Since the medicines did not help much, I came back, got an endoscopy done, and was told that I have a pouch in my foodpipe from where biopsy samples have been sent to rule out cancer. Biopsy results were negative, but my doctor said that suspicion was still there and asked me if I would be willing for a bronchoscopy. Being tired of having gastric issues for a long time, I decided to get all tests done. Bronchoscopy revealed that I had cancer in my foodpipe, which had even spread to my lungs to some extent. My doctor supported me and explained my condition well to me, and told me that an oncologist will also be seeing me. I was given 3 chemotherapy cycles and 30 radiation sessions twice, and then I got a PET scan done. My cancer had reduced, and I was put on some tablets after that. The second PET scan was done in September 2024, and compared to the previous scan, it was not as much of good news. Cancer was still there, and hence, my treatment is still continuing. I would like to thank both my doctors and their whole medical team for providing me with the correct treatment at the right time. It has been two years since my diagnosis, and I hope to defeat my disease.

(Written by the patient in the Kannada language; translated from Kannada to English by the author)
